# Zn-Responsive Proteome Profiling and Time-Dependent Expression of Proteins Regulated by MTF-1 in A549 Cells

**DOI:** 10.1371/journal.pone.0105797

**Published:** 2014-08-27

**Authors:** Wen-jie Zhao, Qun Song, Yan-hong Wang, Ke-jin Li, Li Mao, Xin Hu, Hong-zhen Lian, Wei-juan Zheng, Zi-chun Hua

**Affiliations:** 1 State Key Laboratory of Analytical Chemistry for Life Science, School of Chemistry & Chemical Engineering and Center of Materials Analysis, Nanjing University, Nanjing, PR China; 2 State Key Laboratory of Pharmaceutical Biotechnology, School of Life Science, Nanjing University, Nanjing, PR China; 3 MOE Key Laboratory of Modern Toxicology, School of Public Health, Nanjing Medical University, Nanjing, PR China; Johns Hopkins School of Medicine, United States of America

## Abstract

Zinc plays a critical role in many biological processes. However, it is toxic at high concentrations and its homeostasis is strictly regulated by metal-responsive transcription factor 1 (MTF-1) together with many other proteins to protect cells against metal toxicity and oxidative stresses. In this paper, we used high-resolution two-dimensional gel electrophoresis (2DE) to profile global changes of the whole soluble proteome in human lung adenocarcinoma (A549) cells in response to exogenous zinc treatment for 24 h. Eighteen differentially expressed proteins were identified by MALDI TOF/TOF and MASCOT search. In addition, we used Western blotting and RT-PCR to examine the time-dependent changes in expression of proteins regulated by MTF-1 in response to Zn treatment, including the metal binding protein MT-1, the zinc efflux protein ZnT-1, and the zinc influx regulator ZIP-1. The results indicated that variations in their mRNA and protein levels were consistent with their functions in maintaining the homeostasis of zinc. However, the accumulation of ZIP-1 transcripts was down-regulated while the protein level was up-regulated during the same time period. This may be due to the complex regulatory mechanism of ZIP-1, which is involved in multiple signaling pathways. Maximal changes in protein abundance were observed at 10 h following Zn treatment, but only slight changes in protein or mRNA levels were observed at 24 h, which was the time-point frequently used for 2DE analyses. Therefore, further study of the time-dependent Zn-response of A549 cells would help to understand the dynamic nature of the cellular response to Zn stress. Our findings provide the basis for further study into zinc-regulated cellular signaling pathways.

## Introduction

Cells respond to a variety of extracellular stimuli such as metal stress, growth factors and hormones through the activities of a number of biological processes including metabolism, transcription, development, and differentiation [1]–[3]. The proper functioning of these pathways is disrupted by the deficiency or excess of essential metals or by the presence of other nonessential metals [4], [5]. Zinc, the second most abundant and essential transition metal in human tissues, plays a critical role in regulating vital cellular components such as enzymes and the translation machinery. Zinc is efficiently uptaken from the environment, transported, and stored to maintain intracellular zinc homeostasis. Intracellular zinc ion concentrations are strictly regulated by zinc channels and zinc-binding proteins to maintain cellular zinc-dependent functions [6], [7]. Over 300 enzymes and other proteins have been identified that require zinc for their proper functioning [8], [9]. Zn-binding and Zn-responsive proteins have been extensively identified and assayed by two-dimensional gel electrophoresis (2DE) and mass spectrometry (MS) [10]–[13].

Metal-responsive transcription factor 1 (MTF-1), a zinc finger protein plays an important role in the cellular response to heavy metal stress [14]. This protein functions as a cellular zinc sensor and a critical regulation factor involved in zinc homeostasis [15]–[17]. MTF-1 binds specifically to regulatory DNA sequences called metal response elements (MREs) to activate the expression of genes encoding metallothioneins and zinc transporters in response to zinc ions [18]–[20]. Metallothioneins (MTs) are a family of evolutionarily conserved, low molecular weight, Cys-rich and high-affinity metal-binding proteins [21]. They can sequestrate and release metals, depending on intracellular free zinc concentration, in order to maintain the homeostasis of essential transition metals and protect cells against intracellular oxidative damage [22], [23]. ZIP and ZnT zinc transporters, are also involved in the cellular zinc response [24], [25]. The ZIP family is responsible for transporting zinc ions from the extracellular space or organellar lumen into the cytoplasm. In contrast, ZnT family transporters mediate the efflux of intracellular zinc to prevent the cellular accumulation of the metal ion, thereby limiting the possibility of zinc toxicity [26]. It has been reported that *ZnT-1* transcription is regulated by MTF-1 [27], but the influence of MTF-1 on the expression of *ZIP* family genes is not clear.

The effect of zinc on various biological pathways is highly dependent on its concentration. For example, increased concentration of exogenous zinc may promote aggregation of amyloid-beta peptide in patients with Alzheimer disease [28], [29]. Elevated zinc levels induce cytotoxicity and up-regulate the expression of *ZnT-1* in pancreatic cancer cells [30]. A deficiency of Zn, on the other hand, increases oxidative DNA damage in the prostate [31]. However, little consideration has been paid to time-dependent effects of zinc, particularly in the context of proteome analyses [32], [33]. Moreover, recent discoveries have extended the known functions of MTF-1 in metal homoeostasis beyond regulating the expression of *MT-1* and *ZnT-1* [34]. For example, the transcription of ferroportin 1 (FPN1), the only known iron exporter in vertebrate cells, was induced by zinc through the direct action of MTF-1 binding to the *FPN1* promoter [35]. Unexpectedly, the expression of hepcidin, an inhibitor of FPN1, was also shown to be induced by MTF-1 in a zinc-dependent manner [36]. MTF-1 interacts with some transcriptional co-activators and other stress responsive transcription factors to synergistically maintain zinc homeostasis. In one study, mouse MTF-1 was found to form a zinc-induced complex with the transcription factor Sp1 and the histone acetyltransferase p300 co-activator [37]. In another study, it was suggested that MTF-1 assisted nuclear factor 1 (NF1) with binding to promoter sequences in order to activate gene transcription in a zinc-dependent manner [38].

In order to more fully understand the effect of zinc on different cellular processes, high throughput methods are being employed to detect and characterize more zinc-dependent proteins. For example, an oligonucleotide microarray was used to identify over 100 zinc-responsive genes in zebrafish [39]. A systematic search for *E. coli* proteins with zinc-binding activity was performed using radioactive Zn(II) binding to total *E. coli* proteins fractionated by 2DE [10]. Target genes and interaction partners of MTF-1 orthologues in human, mouse and *Drosophila* have also been searched by the Schaffner group [34]. However, the multiple signaling pathways involving intracellular zinc and the complex effects of zinc on different biological processes have not been thoroughly investigated. Therefore, we attempted to identify as many zinc-responsive proteins as possible and build a network of these proteins to further understand their biological roles and Zn-responsive signaling mechanisms. The high resolving power of 2DE gels allows for the separation of complex protein samples according to isoelectric point, molecular mass, solubility, and relative abundance. Furthermore, 2DE gels can visually reflect changes in protein expression levels, can be used to discern many isoforms and post-translational modifications, and thus offer comprehensive measure of proteins expression [40], [41]. Therefore, we employed a workflow consisting of 2DE combined with protein identification by MS [40].

In this study, using A549 cell line as a key Zn-responsive signal pathway model, we applied a 2DE-MS proteomics approach to profile the global changes in A549 cells in response to extracellular zinc ions. We identified 83 unique zinc-responsive proteins, of which 18 proteins showed a two-fold or more change in abundance. In addition, we investigated time-dependent changes of proteins regulated by MTF-1 in A549 cells in response to extracellular zinc ions by Western blot and RT-PCR technologies. Our results may reflect the time-dependent adaptation of cells to zinc stimulation and provide novel information to further study the mechanisms of zinc-regulated protein expression.

## Materials and Methods

### Chemicals and materials

The human lung adenocarcinoma A549 cell line was purchased from the American Type Culture Collection (ATCC, Manassas, USA). Calf serum, Dulbecco’s modified Eagle’s medium (DMEM) and trypsin were purchased from Thermo Scientific (Waltham, USA). The Cell Counting Kit-8 (CCK-8) was obtained from the Beyot Institute of Biotechnology (Haimen, Jiangsu, China). All other chemicals were obtained as analytical grade reagents from Sigma (St. Louis, MO, USA) or Sinopharm Chemical Reagent (SCRC, Shanghai, China). Chemicals used in 2DE were purchased from Amresco (Ohio, USA) or GE (Piscataway, USA). Water used in experiments was Millipore Milli-Q filtered (Bedford, MA, USA) at a resistivity greater than or equal to 18.25 Ω·cm. Immobilized pH gradient (IPG) strips were purchased from GE (Piscataway, USA). Culture dishes, 6-well plates and 96-well plates were supplied by Costar (Cambridge, USA). Polyvinylidene difluoride (PVDF) membranes were from Millipore (Bedford, MA, USA). Primary antibodies, the MTF-1 rabbit anti-human polyclonal (C-terminus) antibody (LS-C31406/26273), anti-metallothionein antibody (ab12228), anti-SLC39A1 antibody (ab105416), SLC30A1 antibody (NBP1-56273) were purchased. Secondary antibodies consisting of anti-rabbit lgG-HRP (sc-2004) and goat anti-mouse lgG-HRP (sc-2005) were also purchased.

### Cell culture

A549 cells were maintained in DMEM supplemented with 10% calf serum, 100 units/mL penicillin, and 100 mg/mL streptomycin. Cells were routinely incubated at 37°C in a 5% humidified CO_2_-enriched atmosphere. For 2DE and Western blot analysis, cells were grown in 100-mm-diameter culture dishes. Cells were plated in 6-well plates at a density of 1×10^5^ cells/well for RT-PCR, and in 96-well plates at a density of 2×10^3^ to 4×10^3^ cells/well for cell viability analysis. When the cells reached approximately 80% confluency, the cells were harvested.

### Exogenous Zn (II) stress

A stock solution was prepared with ZnSO_4_·7H_2_O. The salt was dissolved in Milli-Q water and sterile-filtered with a 0.22 µm filter. The concentration of zinc was determined to be 85.7 mM using a Perkin-Elmer OPTIMA 5300DV inductively coupled plasma-optical emission spectroscopy (ICP-OES) (MA, USA). A549 cells were treated for different lengths of time or with different concentrations of ZnSO_4_ dissolved in culture medium after cells reached approximately 30% confluency. Fresh culture medium containing 25, 50, 75, 100, 150, 200, 300 or 400 µM ZnSO_4_ was used to treat cells for 24 h or 48 h in cell viability analysis. The cells were harvested and washed twice with 3 mL of pre-warmed (37°C) phosphate-buffered saline (PBS, pH 7.4) to remove any metal contamination derived from the culture medium. A549 cells were treated with a non-physiological concentration of ZnSO_4_ (100 µM) dissolved in culture medium for 24 h for the 2DE experiment. Cells were subsequently incubated with 100 µM ZnSO_4_ for 6, 8, 10, 12, 24 or 48 h for Western blot analyses, and 1, 2, 4, 6, 8, 10, 12, 24 or 48 h for RT-PCR analyses. The control groups were not treated with ZnSO_4_.

### Cell viability assay

CCK-8 was used to test the viability of A549 cells after time-course treatment or exposure to various doses of ZnSO_4_. This assay uses Dojindo’s highly water-soluble tetrazolium salt 2-(2-methoxy-4-nitrophenyl)-3-(4-nitrophenyl)-5-(2, 4-disulfophenyl)-disulfophenyl)-2H-tetrazolium, monosodium salt (WST-8). WST-8 is reduced by dehydrogenases in cells to yield an orange-colored formazan that is soluble in the tissue culture medium, and the amount of the formazan dye is directly proportional to the number of living cells. CCK-8, being nonradioactive, allows for highly sensitive colorimetric assays for the determination of the number of viable cells in cell proliferation and cytotoxicity assays [42]. Approximately 1500 A549 cells in 100 µL culture medium were seeded in each well. The cells were treated with various concentrations of ZnSO_4_ (25, 50, 75, 100, 150, 200, 300 or 400 µM) and incubated for 24 h or 48 h. The medium was renewed with fresh culture medium containing identical concentrations of ZnSO_4_ before assay using the kit. 10 µL CCK-8 solution was added to each well, and the plate was incubated for 30 min to 1 h at 37°C. Absorbance was measured at 450 nm using a BIO-RAD 680 enzyme micro-plate reader (Hercules, CA, USA) after 45 min to obtain stable data.

### Protein sample preparation for 2DE and Western blot

Cells were harvested using ice-cold PBS and collected into 1.5 mL Eppendorf tubes and stored at −80°C. Cell numbers were recorded using a haemocytometer. The number of cells was adjusted to about 1×10^7^ cells per mL in PBS. The cells were lysed by resuspending in 200 µL extraction buffer (7 M urea, 2 M thiourea, 4% w/v 3-((3-cholamidopropyl)dimethyl-ammonio)-1-propanesulfonate (CHAPS), 65 mM DTT, 2% v/v IPG buffer pH 3–10, 0.01% v/v protease inhibitor cocktail and a 0.01% v/v RNAase inhibitor mix) and were vortexed vigorously for 3 min at 4°C. Cell lysates were then sonicated for 1 min at 10% amplitude at interval setting 2 s on and 3 s off with a Sonicator S-4000 (Misonix, Shanghai, China). The heat generated by sonication was below 1000 j. The sample was clarified by centrifugation at 40000×g for 1 h at 4°C. The supernatant was flash-frozen and stored at −80°C. A Bradford protein assay kit was used to quantify proteins at 595 nm. Approximately 8 mg/mL of whole soluble protein for each sample was obtained for 2DE and immunoblot analysis.

### 2DE and image analysis

Separation of proteins was carried out with a GE Healthcare IPGphor IEF and an Ettan Dalt six electrophoresis system. Isoelectric focusing (IEF) was performed with 24 cm precast linear IPG strips (pH 3 to 10). 1 mg of whole cell proteins prepared as above was mixed with 450 µL of rehydration buffer (7 M urea, 2 M thiourea, 2% w/v CHAPS, 18 mM DTT, 0.5% v/v IPG buffer pH 3–10, and 0.002% bromophenol blue (BPB)) and loaded into the IPG strips by in-gel rehydration at room temperature overnight. IEF was performed in a stepwise voltage increase procedure at 20°C with the following parameters: 100 V for 6 h Step (Stp) mode, 500 V for 3 h Stp mode, 1000 V for 1 h Gradient (Grd) mode, 4000 V for 1 h Grd mode, 8000 V for 1 h Grd mode, and 8000 V for 68000 vh Stp mode, 500 V Stp mode holding.

After IEF, the IPG strips were subjected to a two-step equilibration (6 M urea, 30% glycerol, 2% SDS, 0.002% BPB, 75 mM Tris-HCl, pH 8.8) with 1% DTT (w/v) for the first step and 2.5% iodoacetamide (w/v) for the second step. The separation in the second dimension was performed using 1-mm-thick 12% polyacrylamide gels in Tris-glycine buffer (25 mM Tris-HCl, 192 mM glycine, 0.1% SDS, pH 8.3). Electrophoresis was carried out at 2 W/gel for 40 min and then at 15 W/gel for 4 h at 15°C until the BPB marker reached the bottom of the gel.

The gels were then fixed in buffer (40% methanol, 10% acetic acid in Milli-Q water), stained with Coomassie brilliant blue (CBB) R-250, and then destained in the same buffer. The CBB-stained gels were scanned using an ImageScanner (GE Healthcare, Piscataway, USA), and the spot detection and quantification were carried out with ImageMaster 2D platinum 7.0 analysis software (GE Healthcare, Piscataway, USA). Spot detection was conducted using the following threshold setting: smooth of 5, saliency of 150, min area of 200. The number of protein spots was ascertained and a matched set was generated from replicate gels. The average normalized volumes for each spot (% of total spot volume) were compared, and the spots with at least two-fold differential expression between ZnSO_4_ and control groups and an p-value resulting from ANOVA analysis of less than or equal to 0.05 were considered significantly changed, then subsequently subjected to protein identification. All the samples were analyzed in triplicate.

### Protein identification

Protein spots of interest were manually excised from the gels and digested with trypsin (Promega, Sequencing grade, USA). The excised gel pieces were washed with Milli-Q water twice and stored at −80°C. Gel particles were destained with 25 mM ammonium hydrogen carbonate/50% acetonitrile until the particles were colorless, and covered by acetonitrile until gel pieces shrunk. Acetonitrile was removed and gel particles were dried by vacuum centrifugation. Gel particles were incubated with 10 µL of 12.5 ng/µL trypsin in 25 mM ammonium hydrogen carbonate. In-gel digestion with trypsin was performed at 37°C for 20 h. The supernatants from the trypsin-digested mixtures were collected in separate tubes, and peptides were extracted twice using 20 µL 50% acetonitrile/5% formic acid. All supernatants derived from the peptide extracts were mixed, desalted and concentrated using mini-C18 columns (Zip-Tips, Millipore, Bedford, MA, USA).

Extracted peptide samples were analyzed on a 4800 Plus MALDI TOF/TOF Analyzer (Applied Biosystems, Foster City, CA, USA) using 20 kV of acceleration voltage. Each sample was mixed with 10 mg/mL α-cyano-4-hydroxycinnamic acid in 50% acetonitrile and 0.1% trifluoroacetic acid, and was spotted onto MALDI targets. Peptide mass fingerprints (PMF) and MS/MS ions were obtained in positive reflector mode. Proteins were identified using the MASCOT search engine (Matrix Science, London, UK) against the human protein NCBI database using the following search criteria: 50 ppm peptide mass tolerance, 0.1 Da fragment mass tolerance for MS/MS, maximum of 1 missed cleavage, a fixed carbamidomethyl (C) modification of cysteines, and variable oxidation (M).

### Western blot analysis

Whole cell extract proteins were fractionated on 12% acrylamide gels by SDS-PAGE according to Laemmli’s method [43], and proteins were electrotransferred onto PVDF membranes by using a Mini P-4 electrotransfer apparatus (Cavoy, Beijing, China). PVDF membranes were activated by soaking in methanol for 1 min prior to blotting. The membranes were then equilibrated for 10 min in blotting buffer (48 mM Tris-base, 39 mM glycine, 20% (v/v) methanol, and 0.0375 (w/v) SDS). The blotting sandwich was made according to the manufacturer’s instructions. The blotting was carried out for 1 h on ice at constant voltage of 100 V. After transfer, the membrane was blocked in 5% skimmed milk in PBST (PBS containing 0.1% (v/v) Tween-20) for 1∼2 h at room temperature. After four repeated washes with PBST for 4 min, the membrane was incubated with primary antibody overnight at 4°C. Then, the membrane was washed four times with PBST for 4 min and incubated for 1 h at room temperature in the presence of the appropriate horseradish peroxidase-conjugated secondary antibody. After being washed repeatedly, the membrane was incubated with Pierce ECL Western Blotting Substrate (Thermo Scientific, Rockford, USA), and immune complexes were detected using the enhanced chemiluminescence assay (CLINX, Shanghai, China). After adequate development, the reaction was stopped by rinsing with PBST. The low-background membrane was incubated with different antibodies and developed repeatedly.

### RT-PCR

Total RNA was prepared from A549 cells using the TRIzol reagent (Invitrogen, USA) according to the manufacturer’s instructions. RNA samples (1 µg each) were reverse-transcribed as described in the instructions of the PrimeScript RT regent kit (Takara, Japan). 1 µL of the resulting cDNA solution was used for PCR. The genes were amplified in a 20 µL reaction solution using TP 600 (Takara, Japan). After the reaction, reverse transcriptase was inactivated by heating at 94°C for 4 min followed by 29 cycles (for glyceraldehyde-phosphate dehydrogenase, GAPDH, 20 cycles) at 94°C for 30 s, 58°C for 30 s, 72°C for 30 s, and finally a extension step at 72°C for 7 min. A GAPDH primer was used as an internal control and amplifications were quantified in triplicate. Sequences of PCR primers for the analysis of genes of interest are summarized in Table 1. An aliquot (10 µL) of each reaction was analyzed by agarose gel electrophoresis and ethidium bromide staining.

**Table 1:** Nucleotide sequences of primers used for RT-PCR.

		Primer sequences	
Gene	GeneBankaccession No.	Forward	Reverse
*ZIP-1*	NM.014437	GATTGGGGAAGACACTTGACTGCT	GAAAGAGGAAGGGGATTTGTTTGG
*ZnT-1*	NM.021194	GCATCAGTTTATGAGGCTGGTCCT	CAGGCTGAATGGTAGTAGCGTGAA
*MT-1*	NM.005946.2	ATGGACCCCAACTGCTCCTGC	GGCACAGCAGCTGCACTTCTC
*MTF-1*	NM.005955.2	CCACAACACAATGGATCAGAGGA	GAGTTGGCACCCAGGGGCAG

### Statistical analysis

All measurements were repeated a minimum of three times and results were expressed as mean ± standard deviation (SD). Statistical significance for the comparison of two groups was assessed using one-way ANOVA with the Tukey-Kramer multiple comparison post-hoc test. Differences were considered statistically significant at a value of *p*≤0.05.

## Results

### Cell viability

**Figure 1:**
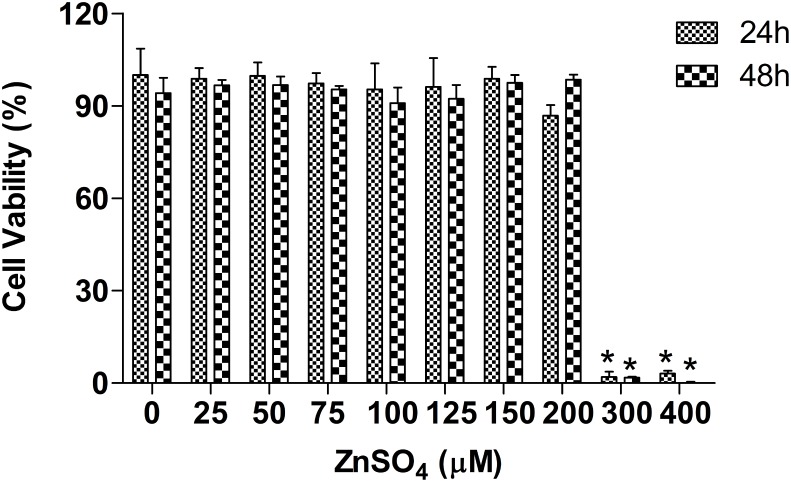
Cell viability analysis of A549 cells after treatment with ZnSO_4_. Cell viability of A549 was assessed using the WST-8 method after treatment with various doses of ZnSO_4_ for 24 h or 48 h. (data are presented as mean ± SD, *p≤0.05 versus controls, n = 3). A sub-cytotoxic metal concentration (100 µM) and exposure time (24 or 48 h) were selected for 2DE, Western blot and RT-PCR analyses.

The metal concentrations used in our experiments were sub-cytotoxic as demonstrated by cytotoxicity assay as shown in Figure 1. The statistical analyses show that the viability of A549 cells was not significantly different at ZnSO_4_ concentrations not more than 200 µM no mater for 24 h or 48 h. Levels of cell death about 10% were observed at ZnSO_4_ concentrations not more than 200 µM for 24 h or 48 h. Increases in ZnSO_4_ concentration led to reduced viability of A549 cells and the viability of A549 cells was lower than 3% after treatment with 300 µM ZnSO_4_ for 24 h or 48 h. In addition, long exposure time length resulted in more cells death at the same exposure concentration. However, the viability of A549 cells was decreased about 10% when treated with 200 µM ZnSO_4_ for 24 h, while treated for 48 h, little sign of cell death was apparent, probably resulting from the great adaptability of the cells in responding to exogenous zinc. Therefore, a ZnSO_4_ concentration of 100 µM for 24 h was selected for 2DE. Sub-cytotoxic metal concentration (100 µM ZnSO_4_) and appropriate exposure times (6, 8, 10, 12, 24 or 48 h for Western blot, and 1, 2, 4, 6, 8, 10, 12, 24 or 48 h for RT-PCR) were used for further analysis.

### 2DE and protein identification

In order to characterize the global effects of exogenous zinc ions on protein expression in A549 cells and obtain more direct information about Zn homeostasis, 2DE coupled with MALDI TOF/TOF was used to detect and identify Zn-responsive proteins. A549 cells have been extensively used in the characterization of metal exposure and in the proteomic analysis of cells [13] [44]. In our 2DE experiments, A549 cells were incubated with 100 µM ZnSO_4_ and harvested after 24 h. This concentration and exposure time and concentration was chosen to ensure that the cells remained approximately 75% confluent without undergoing contact inhibition.

**Figure 2:**
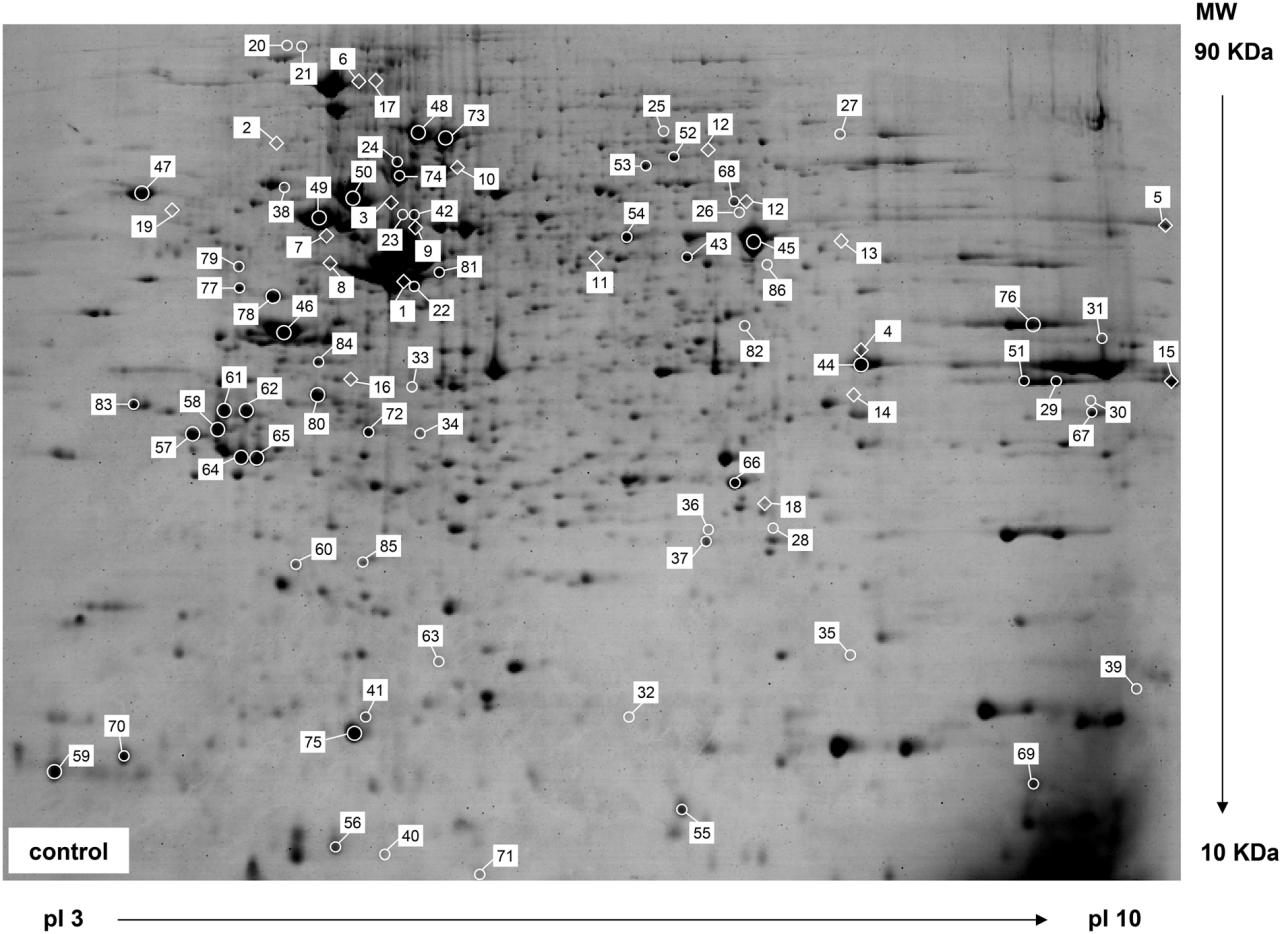
Representative 2DE gel of soluble proteins in A549 cells stained with CBB. Each protein sample (1 mg) was separated by IEF in a 24 cm long IPG gel strip containing a wide-range linear pH gradient of 3–10, followed by SDS-PAGE on a vertical 12% gel. Proteins differentially expressed in response to excess Zn (100 µM ZnSO_4_ for 24 h) are illustrated with a diamond symbol. These proteins showed a two-fold or greater difference in abundance (*p*≤0.05) in A549 cells. Other proteins identified are labeled with a ring symbol.

One milligram of whole cell soluble proteins were separated on a 24 cm precast linear IPG strip (pH 3 to 10) followed by SDS-PAGE. The gels were visualized by CBB staining, which is a simpler and more quantitative method than silver staining, although 50- to 100-fold less sensitive than silver staining. A representative 2DE gel of soluble proteins in A549 cells stained with CBB is shown in Figure 2. ImageMaster 2D Platinum is high-throughput 2DE imaging software for almost parameter-free spot detection. 6361 spots were detected on two groups of parallel gels and 1351 matches were created to reference gels in a MatchSets. 36 proteins were differentially expressed in response to Zn exposure, of which 18 proteins were identified by MS/MS, including 17 Zn down-regulated proteins and 1 up-regulated protein. Descriptions of the 18 differentially expressed proteins identified are presented in File S1 and these proteins are labeled on the representative 2DE gel (Figure 2). The additional 65 unique proteins identified are also listed in File S1 and labeled on the representative 2DE gel (Figure 2).

### Functions of proteins differentially expressed in response to Zn

**Figure 3:**
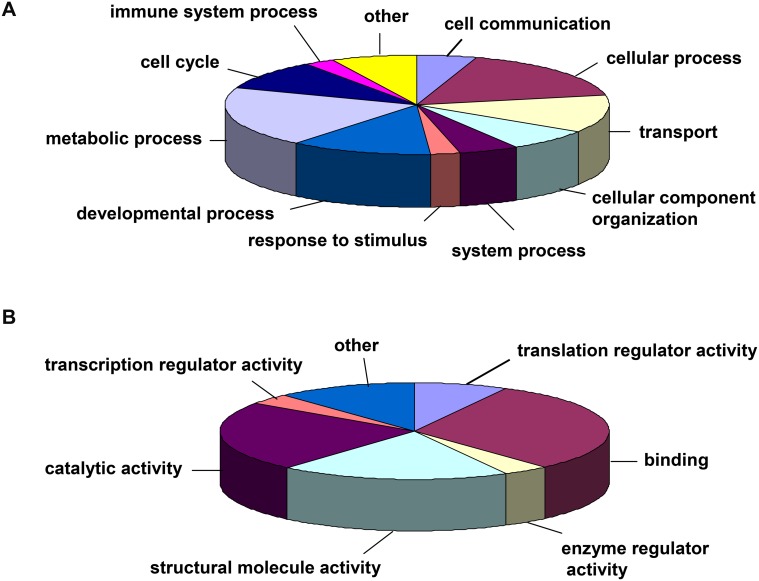
PANTHER classification of proteins that showed differential expression in response to extracellular Zn. (A) Biological process classification; (B) Molecular function classification.

The eighteen proteins identified exhibited significant, reproducible differentially expressed as a result of zinc treatment. They involved in various intracellular physiological activities. Most of them have been previously implicated in various cellular stress responses [13], [32], [45]. The differentially expressed proteins identified were categorized according to the PANTHER Classification System (http://pantherdb.org/). Among these proteins, 15 proteins had a reliable “hit” within the system. They were classified according to biological process (Figure 3A). 11 proteins were predominantly involved in the metabolic process, cellular process, transport process and developmental process categories. Other biological processes accounting for a small percentage of the identified proteins included cell cycle, cellular component organization, cell communication, system process and immune system process categories. Proteins not categorized by PANTHER included RNH1, phosphatase-2A regulatory subunit-beta and beta-enolase isoform. Moreover, the protein hits were classified according to molecular function (Figure 3B), and the descriptions of each molecular function are included in File S1. Binding, catalytic activity and structural molecule activity were the dominant molecular functions of the differentially expressed proteins. Translation regulator activity, enzyme regulator activity and transcription regulator activity accounted for a small number of the total molecular functions obtained. The proteins that did not participate in the PANTHER molecular function categories were annotated according to Uniprot KB. These proteins included HSP90AA1, T-complex protein 1 subunit delta, beta-enolase and guanine nucleotide-binding protein subunit beta-2-like 1. PANTHER classification analysis revealed that there was a great overlap between these molecular functions and biological processes since a protein often possesses multiple functions and participates in multiple biological processes (File S1).

### Western blot analysis

**Figure 4:**
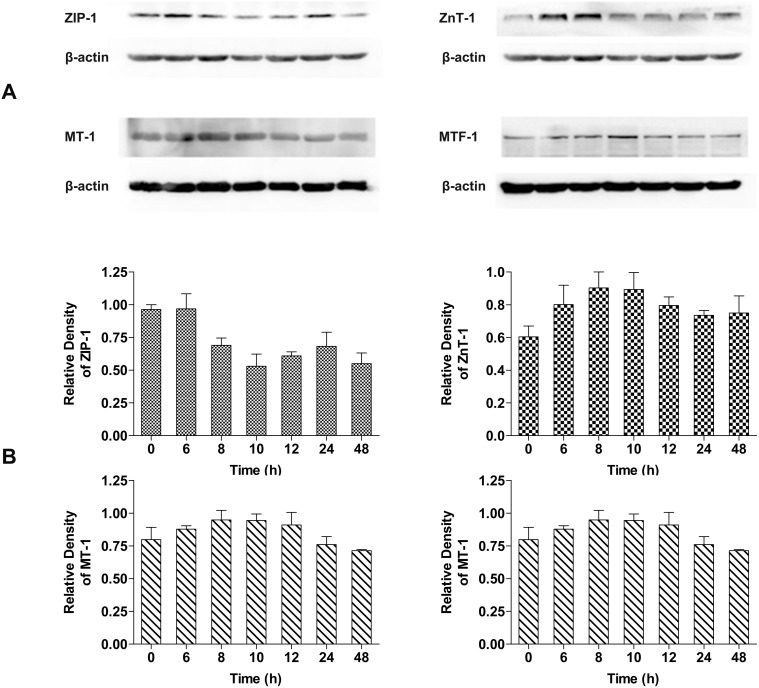
Time-dependent changes in ZIP-1, ZnT-1, MT-1 and MTF-1 abundance in cells treated with ZnSO_4_. (A) Western blot analysis of proteins regulated by MTF-1, including MT-1, ZnT-1 and ZIP-1 in A549 cells after different stimulation periods with exogenous 100 µM ZnSO_4_; (B) Protein band density was analyzed with Gel Image Analysis software (CLINX, Shanghai, China). Beta-actin was used as the internal control. Data was normalized and mean values ± SD were calculated from at least three independent samples.

We initially sought to investigate MTF-1 regulated proteins by 2DE. However, we obtained no evidence on our 2DE gels for those proteins known to be regulated by MTF-1 and thought to be the key proteins in maintaining zinc homeostasis, such as MTs, ZnTs or ZIPs [15], [30], [46], [47]. To explore possible reasons for this, we measured the abundance of influx transporter ZIP-1, efflux transporter ZnT-1, zinc binding protein MT-1 and MTF-1 in A549 cells after treatment with 100 µM ZnSO_4_ in a time-dependent manner. The changes in abundance of these four proteins after 0, 6, 8, 10, 12, 24 and 48 h were analyzed by Western blot as shown in Figure 4. The expression of ZIP-1 sharply decreased to a minimum after 10 h of extracellular Zn exposure, and then increased slowly, but the ZIP-1 level in treated cells was always lower than in untreated cells. ZnT-1 exhibited a converse pattern of expression in that its abundance rapidly increased to a peak at 8 h, and then decreased from 10 h. Unlike ZIP-1, the abundance of ZnT-1 in Zn-treated cells was always higher than in controls. The expression of MT-1 changed in a manner similar to that observed for ZnT-1. The abundance of MT-1 first increased to a plateau between 8 and 10 h, and then decreased. However, the abundance of MT-1 was lower than in controls at 24 h when the abundance of ZnT-1 was higher than in controls. Extracellular Zn application markedly increased the expression of MTF-1 during the first 10 h of exposure. Following this time-point, the abundance of MTF-1 showed a gentle decline until 48 h. These data are in agreement with the reported roles for these proteins in the transportation, storage and sensing of zinc ions [15], [21], [26]. The abundance of these four proteins all showed maximum changes at about 10 h relative to untreated cells. The abundance ratios of ZIP-1, ZnT-1, MT-1 and MTF-1 by Western blot analysis at 10 h of Zn exposure relative to untreated cells were approximately 1.8, 1.5, 1.2 and 1.2, respectively (Figure 4).

### RT-PCR

**Figure 5:**
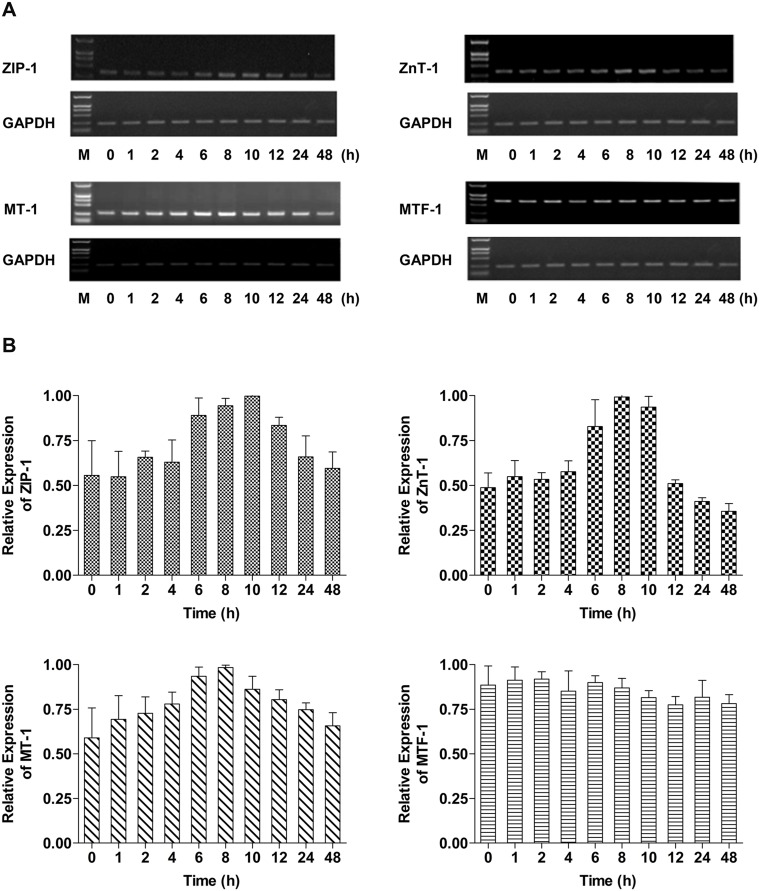
Time-dependent changes in ZIP-1, ZnT-1, MT-1 and MTF-1 transcript abundance in cells treated with ZnSO_4_. (A) RT-PCR analysis of ZIP-1, ZnT-1, MT-1 and MTF-1 mRNA in A549 cells after treatment for 0, 6, 8, 10, 12, 24 and 48 h with exogenous 100 µM ZnSO_4_; (B) Band density was analyzed with Gel Image Analysis software (CLINX, Shanghai, China). GAPDH expression was used as the internal control. Data was normalized and mean values ± SD were calculated from at least three independent samples.

Time-dependent alterations in the mRNA levels of genes encoding ZIP-1, ZnT-1, MT-1 and MTF-1 in A549 cells exposed to 100 µM ZnSO_4_ were analyzed at 0, 1, 2, 4, 6, 8, 10, 12, 24 and 48 h, and the results are shown in Figure 5. ZIP-1 mRNA levels remained stable for 4 h, were up-regulated by 6 h and achieved their maximum level at 10 h after treatment. Following this time-point, ZIP-1 mRNA gradually decreased to control levels. Following stable mRNA levels for 4 h, ZnT-1 mRNA showed a sharp increase from 6 to 10 h after Zn exposure. Subsequently, ZnT-1 mRNA levels decreased to levels below those observed in untreated controls at 24 h. Abundance of MT-1 mRNA levels in response to Zn treatment exhibited a similar pattern to ZIP-1 mRNA levels. However, expression of the gene encoding MT-1 was down-regulated relatively gradually but maintained a higher level of expression than in control cells at 48 h. Of the four genes studied by RT-PCR, expression of *MTF-1* was least influenced by exogenous Zn treatment.

## Discussion

WST-8 analysis indicated that A549 cells exhibit a strong tolerance to ZnSO_4_, and the viability of cells was not decreased significantly even after treatment with 200 µM for 24 h as well as 48 h. However, the cell viability treated with 200 µM for 48 h looked like a little more than that for 24 h, which was possible because the adaptive response of cells was induced by Zn-exposure after 48 h in order to maintain the homeostasis of intracellular zinc.

A total of 86 protein spots in 2DE gels were identified and quantified by MALDI TOF/TOF followed by a MASCOT search. Many of the identified proteins were present as multiple isoforms. For example, four isoforms of elongation factor 1 (alpha, beta, gamma, delta), three isoforms of T-complex protein 1 subunit (delta, zeta, eta) and three isoforms of 14-3-3 protein (beta, epsilon, zeta) were identified, each characterized by a different pI and molecular weight (File S1). These multiple isoforms are the result of transcription from distinct loci or from alternative splicing of the same transcript [48], which causes clusters or lines of spots on the 2DE gels. In addition, the particular ability of 2DE to separate proteins with small changes in their pI allows efficient investigation of modified proteins. Some proteins are post-translationally regulated, such that one or more amino acid residues are modified after the translation process [41]. Proteins modified post-translationally were detected as chains of spots horizontally across the 2DE gels. Although we did not further investigate proteins presenting as multiple spots, some of them were likely involved in the cellular Zn response and MTF-1 regulation [41]. The particular merits of 2DE gels offer a unique perspective on the zinc-responsive proteome.

Eighteen proteins were unequivocally identified by MS/MS, and exhibited significant changes in abundance when A549 cells were exposed to 100 µM ZnSO_4_ for 24 h. Interestingly, the majority of these proteins were down-regulated in response to extracellular Zn, which suggests that inhibition of the expression of many proteins may be a predominant effect of Zn stress on A549 cells. Hogstrand group [39] also reported a similar inhibitory effect on gene expression in the presence of zinc ions by oligonucleotide array. Proteins differentially expressed in response to extracellular Zn are involved in a variety of biological processes and molecular functions. Catalytic activity and structural molecule activity were the dominant molecular functions of Zn-responsive proteins identified in our study and the majority of these were also grouped into the metabolic process, cellular process, transport process and developmental biological process categories. These results provide an overview of the molecular functions and biological processes associated with proteins differentially expressed in A549 cells exposed to zinc ions.

In our proteomics study, none of the zinc-related proteins reported to be regulated by MTF-1 displayed significant differential expression. Our initial purpose for conducting 2DE proteome experiments was to search for novel Zn-responsive proteins and to better understand the signaling network involving the MTF-1 response to Zn. Difficulties in detecting known MTF-1 regulated proteins by 2DE may be attributed to the limitations of this method to visualize or detect proteins that are only weakly expressed. It is also possible that the cellular response to zinc ions is a dynamic process that requires fine temporal control over the alternate expression of many proteins. To test this hypothesis, we conducted additional Western blotting experiments to monitor the expression of known proteins regulated by MTF-1 during several time intervals after extracellular Zn exposure. Expression of the influx transporter ZIP-1 reached minimum levels at 10 h after Zn exposure, while expression of the efflux transporter ZnT-1, as well as MT-1 and MTF-1 increased to their maximum levels at approximately 10 h. These results are consistent with the reported functions of these proteins in maintaining the homeostasis of zinc. ZIP family proteins uptake extracellular zinc ions, and MTF-1 can be activated by zinc ions and induce the transcription of MT-1 which tightly binds zinc and proteins of the ZnT family which export excess zinc out of the cell or into zincosomes [34], [49], [50]. The most dramatic changes in the abundance of these proteins were observed at approximately 10 h following extracellular Zn treatment. After 10 h of exposure, their abundance gradually returned to control levels. For each of these proteins, the differences between maximum and minimum levels of expression observed were less than two-fold. Therefore, it is not surprising that the differential expression of proteins regulated by MTF-1 was not detected by 2DE proteome profiling, because these proteins displayed only slight changes in abundance after only 24 h of Zn treatment.

When exposed to exogenous zinc ions, cells begin to rapidly cycle by increasing expression of zinc efflux transporters and the zinc-binding proteins and decreasing expression of zinc influx transporters. The high affinity of transcription factors like MTF-1 for zinc would allow them to function as “toggle switches” alternating between zinc deficient and replete states [26]. In our experiments, zinc ions stress induced the expression of the efflux transporter ZnT-1 at 6 h after treatment, and the zinc-binding protein MT-1 began to be synthesized at the same time, while the expression of the influx transporter ZIP-1 remained unchanged. At 8 h after Zn treatment, zinc-responsive changes in the expression of the influx transporter ZIP-1 also took place, because the cells need to transport less zinc ions in order to maintain a proper concentration of intracellular zinc. With the accumulation of zinc ions, the A549 cells were at a critical point of physiological equilibrium at 10 h after treatment, and the expression of ZnT-1 reached a maximum. Simultaneously, ZIP-1 and MT-1 coordinated with ZnT-1 to maintain zinc homeostasis in A549 cells. Maximum changes in the abundance of these three proteins indicate an adaptive response to exogenous zinc ion exposure at this time-point. After 10 h, efflux of zinc ions gradually decreased and ZnT-1 expression also fallen slowly until the 48 h time-point. The accumulated deficiency of zinc ions then began Zn influx by modestly increasing expression of ZIP-1. The expression of ZIP-1 was lower than in untreated control cells due to the excess of zinc ions. However, the expression of MT-1 and ZnT-1 was higher than in untreated control cells. The expression of MTF-1 acted as a switch and alternated with the efflux and influx of zinc ions. Therefore, at the 10 h time-point, the proteome of A549 cells probably exhibited more significant changes owing to the time-dependent adaptive response of A549 cells to ZnSO_4_.

The expression of MTF-1, ZnT-1 and MT-1 transcripts was similar to the patterns observed for their corresponding protein levels, but peaks seemed to occur approximately two hours earlier (i.e. at 8 h after Zn exposure). This is likely due to the more rapid synthesis of mRNA than protein. Unexpectedly, we observed that the abundance of ZIP-1 transcripts displayed an opposite tendency compared to the protein level, which may be due to a complex mechanism of ZIP-1 regulation. ZIP-1 may be involved in multiple signaling pathways, not only the MTF-1-regulated Zn-responsive pathway, but also other biological processes. The disparity between the levels of mRNA and their corresponding proteins may occur because of post-transcriptional and post-translational modifications, as well as differential mRNA and protein degradation rates and other biological processes [51]–[54].

## Conclusions

In summary, this work has provided an overview of the proteome profiles of A549 cells in response to exogenous zinc ion stress *in vivo*. Most differentially expressed proteins in response to zinc ions are involved in metabolic process, cellular process, transport process and developmental process categories. Due to the limitations of 2DE proteome technology, we did not detect differentially expressed proteins known to be regulated by MTF-1 and involved in zinc homeostasis. However, our findings contributed proteome-related information that will be useful in understanding the mechanisms of Zn-responsive proteins and protein expression regulated by MTF-1 in A549 cells. In addition, the functions of some of the identified proteins could be related to the Zn response or signaling mechanisms regulated by MTF-1, although the functional significance of our detected proteins remains unclear and needs further investigation. More importantly, we investigated the time-dependent changes in the expression of proteins regulated by MTF-1, including ZIP-1, ZnT-1 and MT-1. These proteins exhibited maximum changes in their expression at approximately 10 h following Zn treatment, and the proteome of A549 cells perhaps will exhibit significant changes originated from time-dependent adaptive response to exogenous zinc ions exposure. Therefore, we may obtain more significant and comprehensive information concerning changes in the proteome of A549 cells in response to Zn stress by employing 2DE or other technologies in a time-dependent manner.

## Supporting Information

File S1 **Proteins identified by MALDI TOF/TOF.**
(XLS)
